# GATD3A-mediated monocyte homeostasis and compartmentalized erythrocyte alpha-synuclein discriminate early Parkinson’s disease from MSA-P

**DOI:** 10.3389/fimmu.2026.1818028

**Published:** 2026-07-09

**Authors:** Ying Jiang, Chengcheng Xu, Jianing Jin, Xin Liu, Huihui Cai, Wanyu Zhang, Xiaoqing Zheng, Jiayi Wu, Zhan Wang, Yingshan Piao, Tao Feng

**Affiliations:** 1 Center for Movement Disorders, Department of Neurology, Beijing Tiantan Hospital, Capital Medical University, Beijing, China; 2 China National Clinical Research Center for Neurological Diseases, Beijing, China; 3 Department of Child and Adolescent Mental & Behavioral Health, Mental Health Center, Fifth People’s Hospital, ZiBo, Shandong, China; 4 Department of Neurology, Affiliated Hospital of Qingdao University, Qingdao, China; 5 Nursing Department of Beijing Tiantan Hospital, Capital Medical University, Beijing, China; 6 Capital Medical University, Beijing, China

**Keywords:** classical monocytes, multiple system atrophy, Parkinson’s disease, peripheral immune cells, single-cell RNA sequencing

## Abstract

**Background:**

Differentiating early-stage Parkinson’s disease (PD) from the parkinsonian variant of multiple system atrophy (MSA-P) remains a significant challenge due to overlapping clinical phenotypes. This study aimed to identify novel, minimally invasive biomarkers by integrating high-resolution peripheral monocyte transcriptomics with erythrocytic alpha-synuclein (alpha-Syn) distribution patterns.

**Methods:**

We recruited 149 participants (75 PD and 74 MSA-P) to evaluate clinical features and composite peripheral inflammatory indices. To resolve subtle immune heterogeneity often masked in bulk blood analysis, single-cell RNA sequencing (scRNA-seq) was performed on peripheral blood mononuclear cells (PBMCs) from early-stage patients, followed by Quantitative Real-Time PCR (qRT-PCR) validation. Additionally, immunofluorescence was utilized to characterize the subcellular compartmentalization of alpha-Syn forms in erythrocytes.

**Results:**

While MSA-P patients exhibited more rapid progression and severe autonomic failure, standard inflammatory markers (e.g., NLR, MLR) failed to distinguish PD from MSA-P in the early clinical stages. However, scRNA-seq revealed distinct monocyte landscapes. Early MSA-P monocytes displayed an aggressive pro-inflammatory profile driven by chemokine upregulation (CXCL10, CXCL5), interferon pathway activation (IFI27). Conversely, early-stage PD monocytes exhibited a profile of precise homeostatic regulation and protective mitochondrial adaptation, characterized by significant GATD3A downregulation verified by qRT-PCR. Furthermore, distinct pathological patterns were identified: oligomeric alpha-Syn was predominantly sequestered on erythrocyte membranes in MSA-P, whereas pS129 alpha-Syn localized primarily to the cytoplasm in PD.

**Conclusion:**

This study uncovers a divergent peripheral immune landscape where GATD3A-mediated monocyte homeostasis distinguishes early PD from the chemokine-driven inflammation and intense oxidative stress of MSA-P. Combined with distinct erythrocytic alpha-Syn membrane distribution patterns, these signatures offer a promising multidimensional strategy for the early differential diagnosis of these synucleinopathies.

## 1 Introduction

Parkinson’s disease (PD) is a prevalent neurodegenerative disorder pathologically characterized by the abnormal aggregation of pathogenic α-synuclein (α-syn) and the progressive loss of dopaminergic neurons in the substantia nigra (1). Multiple System Atrophy (MSA), clinically manifesting with parkinsonism, cerebellar ataxia, and autonomic failure, is classified into two subtypes: the parkinsonian variant (MSA-P) and the cerebellar variant (MSA-C) (2). Its hallmark pathological feature is the accumulation of aberrant α-syn within oligodendroglial cytoplasmic inclusions (3). Given that MSA exhibits a significantly more rapid clinical progression and poorer prognosis compared to PD, elucidating the distinct pathophysiological mechanisms underlying these two synucleinopathies is critical. Such insights are essential for identifying reliable biomarkers for early differential diagnosis and developing precise therapeutic interventions.

Peripheral biofluids—including whole blood (4), cerebrospinal fluid (CSF) (4), and plasma (5)—have emerged as promising reservoirs for diagnostic biomarkers. In particular, erythrocytes (red blood cells, RBCs) have been reported to harbor elevated levels of alpha-syn forms in patients with PD and MSA compared to healthy controls (6, 7). Previous research indicates that erythrocytes and substantia nigra dopaminergic neurons share similar SNCA transcriptional regulators, potentially leading to parallel elevations in alpha-syn levels (8). A recent study further revealed that, compared to PD, patients with MSA exhibit greater deposition of phosphorylated α-Syn with a more widespread peripheral distribution (9) Consequently, directly observing differences in the distribution of α-Syn within peripheral erythrocytes may offer a window into the divergent progression trajectories of these synucleinopathies.

Research has demonstrated that neurotoxic pathological α-syn fibrils can activate central astrocytes and microglia, inducing central nervous system (CNS) inflammation, which is intimately linked to the pathogenesis and progression of PD (10). Historically, the CNS was viewed as an “immune-privileged site” (11); however, recent findings indicate that an inflammatory milieu can alter blood-brain barrier (BBB) permeability, facilitating peripheral immune cell infiltration (12). These infiltrating cells, in concert with resident innate immune cells, perpetuate neurodegeneration. Thus, peripheral immune dysregulation is increasingly recognized as an active driver of disease progression rather than a mere bystander. For instance, a meta-analysis of 377 PD patients and 355 controls confirmed a significantly elevated neutrophil-to-lymphocyte ratio (NLR) in PD (13), suggesting that peripheral profiles largely mirror the CNS inflammatory state.

Current investigation into peripheral immunity in parkinsonism focuses primarily on lymphocytes, monocytes/macrophages, and granulocytes (14). Indices such as NLR, monocyte-to-high-density lipoprotein ratio (MHR), and red blood cell distribution width-to-platelet ratio (RPR) have served as novel biomarkers for systemic inflammation (15). Monocytes, as key effectors of innate immunity, exhibit high plasticity and heterogeneity: classical monocytes (pro-inflammatory) can differentiate into intermediate monocytes (antigen-presenting) and non-classical (patrolling) subsets (16). Notably, infiltrating CD163+ macrophages have been detected in the peripheral blood and brain tissue of PD patients, as well as in 6-hydroxydopamine (6-OHDA)-induced PD animal models (17, 18). As a monocyte-specific biomarker, CD163 has been shown to correlate with α-syn, tau protein, and tau phosphorylation (19). This collectively underscores the pivotal role of monocytes in PD progression.

Regarding MSA, prior studies have found significantly elevated levels of monocyte chemoattractant protein-1 (MCP-1)—a molecule involved in monocyte recruitment—in the CSF of patients (20). Jiang et al. demonstrated that MHR levels were significantly higher in MSA than in PD, indicating a more severe inflammatory profile (21). Other studies propose monocytes, alongside NLR, as potential diagnostic markers for MSA (22). These findings further imply that peripheral neuroinflammation in MSA parallels central pathological changes, with monocytes acting as significant participants. However, there is a paucity of research specifically dissecting peripheral monocyte-related indices to differentiate early-stage PD from MSA-P, warranting in-depth exploration.

Single-cell RNA sequencing (scRNA-seq) provides unprecedented resolution to resolve immune heterogeneity masked by bulk analysis. The diversity and plasticity of immune cells are fundamental to maintaining homeostasis, and their dysfunction is often subtle and subset-specific. Leveraging scRNA-seq allows for the capture of dynamic gene expression profiles to identify pathogenic mechanisms. For example, Xiong et al. identified specific immune subsets and correlated XCL2 expression in NK cells with PD severity (23). Nevertheless, whether distinct gene expression profiles exist in peripheral monocytes between early PD and MSA-P, and how these differences influence disease trajectories, mains to be elucidated.

Therefore, this study aims to: (1) Investigate potential differences in peripheral blood composite inflammatory indices between patients with possible MSA-P and early-stage PD; (2) Compare the distribution characteristics of α-Syn forms in peripheral erythrocytes between MSA-P and early PD to assess their diagnostic potential; and (3) Utilize scRNA-seq and quantitative PCR to deeply mine differences in gene expression patterns, functional pathways, and intercellular communication of peripheral monocytes between early-stage PD and possible MSA-P. This approach seeks to provide a basis for elucidating the divergent pathological processes of these synucleinopathies and to identify potential targets for early intervention.

## 2 Materials and methods

### 2.1 Subjects and ethics statement

This study was approved by the Ethics Committee of Beijing Tiantan Hospital (KY2022-190-03) and conducted in strict accordance with the Declaration of Helsinki. All participants provided written informed consent. We recruited participants from the Department of Neurology at Beijing Tiantan Hospital, Capital Medical University. PD diagnoses were based on the Movement Disorder Society (MDS) Clinical Diagnostic Criteria (24), with early-stage PD defined as a Hoehn and Yahr (H&Y) stage ≤2.5 (25, 26). MSA diagnoses adhered to the second consensus criteria (2008). Diagnoses were confirmed by two experienced neurologists specializing in neurodegenerative diseases.

The study was conducted in three phases:

Clinical Cohort: Between December 2019 and August 2025, 74 patients with probable or possible MSA and 75 patients with PD were enrolled for clinical and peripheral inflammatory index evaluation. Concurrently, a subgroup analysis of 36 early-stage PD and 38 possible MSA-P patients was performed to determine if these inflammatory markers could distinguish the two conditions at early disease stages.

scRNA-seq Cohort: Between November 2024 and May 2025, 3 patients with possible MSA, 3 with early-stage PD, and 3 healthy controls (HCs) were recruited. PBMCs were collected to characterize immune cell subsets and differential gene expression.

Validation Cohort: Between July 2025 and January 2026, an independent cohort of 6 possible MSA and 5 early-stage PD patients was enrolled for qRT-PCR validation of monocyte gene expression. Exclusion criteria for all participants included significant somatic comorbidities (e.g., tumors, hypertension, diabetes, renal disease, autoimmune disorders), psychiatric conditions (e.g., dementia), and a family history of hereditary neurological diseases. Age-matched HCs were free of neurodegenerative, neurogenetic, or movement disorders.

### 2.2 Evaluation and data collection

Demographic data and clinical characteristics were collected for all participants. Evaluations included disease duration, renal function, medical history, neurological examinations, levodopa equivalent daily dose (LEDD), and sonographic post-void residual (PVR) urine volume measurements. PD patients were further assessed using the Unified Parkinson’s Disease Rating Scale (UPDRS)-III and H&Y staging. Non-motor symptoms were evaluated via the supine-to-standing test (STS) for orthostatic hypotension, Mini-Mental State Examination (MMSE) and Montreal Cognitive Assessment (MoCA) for cognition, and assessments for constipation and rapid eye movement sleep behavior disorder (RBD). Peripheral blood samples were collected from participants free of acute infection or systemic inflammation. Routine blood counts and biochemical indices were measured using an automatic hematology analyzer (Sysmex XN-20A1, Kobe, Japan) and a biochemical analyzer (Beckman AU5800, Brea, CA, USA). Inflammatory ratios were calculated as follows: NLR (neutrophil-to-lymphocyte ratio), LMR (lymphocyte-to-monocyte ratio), MHR (monocyte-to-HDL cholesterol ratio), PLR (platelet-to-lymphocyte ratio), NMR (neutrophil-to-monocyte ratio), and RPR (red cell distribution width-to-platelet ratio). All patients underwent brain magnetic resonance imaging (MRI) to assist in differential diagnosis.

### 2.3 Erythrocyte collection and immunofluorescence

Venous blood (5 mL) was collected from 10 MSA-P and 10 PD patients into K2-EDTA Vacutainers (BD, Franklin Lakes, NJ, USA) and processed within 2 h. Red blood cells (RBCs) were isolated from whole blood by centrifugation at 1,500 × g for 10 min at 4 °C.

The RBC pellet was washed with phosphate-buffered saline (PBS) and centrifuged at 3,000 × g for 10 min at 4 °C. The supernatant was discarded, and washed RBCs were diluted in PBS. Smears were prepared using a cytospin (700 rpm for 6 min), fixed with methanol for 20 min at room temperature, and permeabilized with 0.1% Triton X-100.After blocking with 5% bovine serum albumin (Sigma, Poole, UK) for 1 hour, slides were incubated overnight at 4 °C with primary antibodies: MJFR1 (1:200, Abcam) for total α-Syn, MJFR-14-6-4-2 (1:200, Abcam) for oligomeric α-Syn, and anti-pS129 α-Syn (1:200, BioLegend). Slides were rinsed and incubated for 1.5 hours at room temperature with Alexa Fluor-conjugated secondary antibodies. Finally, the Zenon™ Alexa Fluor 405 Mouse IgG1 Labeling Kit (Life Technologies) was used to label CD235a on the erythrocyte membrane. Images were acquired using a Zeiss LSM 700 confocal microscope with a 40×objective.

### 2.4 PBMC dissociation and scRNA-seq library preparation

Peripheral blood mononuclear cells (PBMCs) were isolated within 4 hours of collection using LeucoSep tubes prefilled with Ficoll-Paque Plus (centrifugation at 700 × g, 20 min). Isolation and subsequent scRNA-seq experiments were performed at Cosmos Wisdom Biotech Co., Ltd. After collecting the PBMCs from the interphase layer, remaining erythrocytes were lysed with red blood cell lysis buffer (Miltenyi Biotec). The cells were washed twice to remove ambient RNA, then resuspended in 1X PBS with 0.04% bovine serum albumin. To ensure a single-cell suspension, samples were passed through a 30-µm Flowmi Cell Strainer. Only cell suspensions with viability exceeding 90% were used for subsequent processing.

### 2.5 Single-cell sequencing

Single-cell RNA sequencing (scRNA-seq) libraries were prepared using the 10x Genomics Chromium Controller Instrument and the Chromium Single Cell 3’ V3.1 Reagent Kits. Briefly, cells were concentrated to 1,000 cells/µL, and approximately 20,000 cells were loaded into each channel to generate single-cell Gel Bead-In-Emulsions (GEMs), resulting in expected mRNA barcoding of 10,000 single cells for each sample. After reverse transcription, GEMs were broken, and barcoded cDNA was purified and amplified. The amplified barcoded cDNA was fragmented, A-tailed, ligated with adaptors, and index PCR amplified. The final libraries were quantified using the Qubit High Sensitivity DNA assay (Thermo Fisher Scientific), and the size distribution was determined using a High Sensitivity DNA chip on a Qsep100 (Bioptic). All libraries were sequenced on an Illumina NovaSeq 6000 platform with a 150 bp paired-end run.

### 2.6 Single-cell RNA statistical analysis

Data analysis was conducted using the Cosmos Wisdom scVision Cloud Analysis Platform. Raw reads were filtered using fastp (27) and aligned to the human genome (GRCh38-2020-A) via Cell Ranger v7.1.0. Down-sampling was applied to normalize sequencing depth across samples. Low-quality cells (<200 genes or >10% mitochondrial UMI counts) were excluded.

The Scanpy package (version 1.9.3) was used for cell normalization and regression based on the expression table, according to the UMI counts of each sample and percentage of mitochondria, to obtain scaled data. PCA was constructed based on the scaled data with all highly variable genes (top 2000), and the top 30 principal components were used for tSNE and UMAP construction. Utilizing a graph-based clustering method, we acquired unsupervised cell cluster results based on the top 30 PCA components. Marker genes were calculated using the scanpy.tl.rank_genes_groups function with the Wilcoxon rank-sum test algorithm under the following criteria: Log2 FC>0.5, pval_adj<0.05, and pct_nz_group>0.25. For detailed cell type identification, subsets of clusters were selected for re-UMAP analysis, graph-based clustering, and marker analysis.

### 2.7 Differential gene expression and GSVA

Differentially expressed genes (DEGs) between groups were identified using the FindMarkers function (Wilcoxon rank-sum test; Log2FC > 0.25, adjusted p < 0.05). Pathway activity per cell type was scored using Gene Set Variation Analysis (GSVA, package v2.0.4) to estimate pathway variation in an unsupervised manner.

### 2.8 Total RNA extraction and quantitative real-time PCR

Total RNA was extracted from monocytes using RNAiso Plus (Takara) and stored at -80 °C. cDNA was synthesized using the PrimeScript™ RT Master Mix (Takara). Quantitative Real-Time PCR (qRT-PCR) was performed on an Applied Biosystems QuantStudio™ 3 System using primers listed in **Supplementary Table 1**. qRT-PCR was performed using GAPDH as the internal reference gene. Relative gene expression levels were calculated via the 2^−ΔΔCt method, with all samples normalized to GAPDH expression. Data analysis was performed using GraphPad Prism (v10.1.2).

### 2.9 Statistical analysis

Sequencing data were analyzed using R software (Version 4.4.1) and Cell Ranger (7.2.0). All data are presented as primary data. Statistical analysis was performed using SPSS Statistics 25.0 (IBM, Chicago, IL, USA). Continuous variables with normal distribution were presented as mean ± standard deviation (SD), and those with non-normal distribution as median (interquartile range). The t-test and chi-square test were used to compare differences in clinical data between groups. The Mann-Whitney U test was used for non-normally distributed data between two groups. A two-sided *P* < 0.05 was considered statistically significant. Several R packages were utilized: Venn Diagram for generating Venn diagrams; Seurat R package’s FindMarkers for identifying DEGs; and pheatmap R package for heatmaps.

## 3 Results

### 3.1 Demographic and clinical characteristics of the study cohorts

To investigate the clinical distinctions between MSA-P and PD, we recruited 149 participants: 74 with MSA-P and 75 with PD. Baseline demographic and clinical characteristics are summarized in **Table 1**.

**Table 1:** Demographic and clinical characteristics of the total study cohort (MSA-P vs. PD).

Characteristics	MSA-P(n=74)	PD(n=75)	P
Females/Males	47/27	35/40	**0.039**
Age, years	62.00(57-67)	64.00(57-67)	0.33
Duration, years	3.08 ± 1.60	6.64 ± 3.75	**<0.001**
Leukocyte count(x 10^9^/L)	5.77(4.95-6.71)	5.59(5.06-7.18)	0.485
Neutrophils(x10^9^/L)	3.61 ± 1.31	4.16 ± 2.03	0.051
Lymphocytes(x10^9^/L)	1.62(1.29-2.17)	1.54(1.20-1.84)	0.059
Eosinophils(x10^9^/L)	0.14 ± 0.12	0.12 ± 0.11	0.140
Basophils (x 10^9^/L)	0.03 ± 0.02	0.03 ± 0.02	0.830
Monocytes (x 10^9^/L)	0.33(0.27-0.42)	0.37(0.27-0.44)	0.372
PLT (x 10^9^/L)	208.35 ± 52.54	218.80 ± 65.82	0.286
MPV (fl)	9.50(8.90-10.10)	9.50(8.80-10.10)	0.732
RDW	41.25(40.00-43.00)	41.10(40.30-43.60)	0.574
UA	280.75(229.38-321.28)	284.30(230-341.90)	0.628
PVR urine volume, ml	102.30 ± 138.86	15.99 ± 28.58	**<0.001**
HDL-C	1.28(1.13-1.44)	1.22(1.06-1.52)	0.884
Hcy	14.78 ± 7.30	15.65 ± 8.54	0.508
MMSE	25.74 ± 3.69	26.43 ± 3.91	0.274
Moca	21.50(17.00-26.00)	22.00(19.00-25.00)	0.813
1minΔSBP,mmHg	16.00(6.50-28.00)	4.00(-6.00-13.00)	**<0.001**
1minΔDBP,mmHg	4.00(-1.00-10.00)	-0.00(-9.00-4.00)	0.180
1minΔHR	-6.00(-11.00--3.00)	-6.00(-11.00--1.00)	0.513
3minΔSBP,mmHg	16.97 ± 16.51	5.43 ± 15.94	**<0.001**
3minΔDBP,mmHg	5.00(-1.00-10.00)	-2.00(-7.00-3.00)	**<0.001**
3minΔHR	-6.00(-10.00--2.00)	-6.00(-10.00--1.00)	0.626
eGFR,ml/min	108.40(103.14-114.04)	107.21(100.98-113.97)	0.933
Constipation%	83.78	58.67	**0.003**
diabetes%	10.81	20.00	0.053
nephropathy%	5.41	16.00	**0.037**
hypertension%	27.03	33.33	0.402
RBD%	78.38	44.00	**<0.001**
smoking%	20.27	26.67	0.357
Drinking%	24.32	22.67	0.811
LEDD,mg	179.45(161.08-207.75)	800.00(600.00-1087.00)	**<0.001**
Duration(ms)	13.00(11.90-13.90)	10.90 (10.30-12.10)	**<0.001**
Polyphasity(%)	47.61 ± 20.84	40.26 ± 18.39	**0.025**
Percentage of satellite potentials(%)	10.00(5.00-15.00)	4.85(0.00-5.00)	**0.001**

PVR, post-void residual; LEDD, levodopa equivalent daily dose; MSA, Multiple system atrophy; PD, Parkinson’s Disease; HDL-C: high-density lipoprotein cholesterol; Hcy: homocysteine; ΔSBP: changes in systolic blood pressure during the supine-to-standing test. ΔDBP: changes in diastolic blood pressure during the supine-to-standing test; MMSE, Mini-Mental State Examination; MoCA, Montreal Cognitive Assessment.

Comparison of baseline demographic variables, clinical scales, and autonomic parameters between patients with Multiple System Atrophy-Parkinsonian variant (MSA-P, n=74) and Parkinson’s Disease (PD, n=75). Data are shown as the mean (SD) for normally distributed continuous variables, median (IQR) for nonnormally distributed continuous variables, and n (%) for categorical variables. The t-test and the chi-square test were used to compare differences in clinical data between groups. The Mann–Whitney U test was used for non-normally distributed data between two groups. A two-sided p-value < 0.05 was considered statistically significant.

Bold values indicate statistically significant differences.

There were no statistically significant difference in age between the two groups (P = 0.33). However, MSA-P patients had a significantly shorter disease duration (3.08 ± 1.60 years) compared to the PD cohort (6.64 ± 3.75 years; P<0.001), reflecting the rapid progression characteristic of MSA-P.

Regarding autonomic parameters, MSA-P patients exhibited severe urogenital failure, evidenced by significantly higher PVR urine volume (102.30 ± 138.86 ml) compared to the PD group (15.99 ± 28.58 ml)(P<0.001). Cardiovascular autonomic neuropathy was also more pronounced in the MSA-P group. Upon orthostatic challenge, the drop in systolic blood pressure (ΔSBP) was significantly greater in MSA-P patients at both 1-minute (16.00 [6.50–28.00] mmHg vs. 4.00 [-6.00–13.00] mmHg; P<0.001) and 3-minute intervals (16.97 ± 16.51 mmHg vs. 5.43 ± 15.94 mmHg; P<0.001). Similarly, the drop in diastolic blood pressure (ΔDBP) at 3 minutes was significantly larger in the MSA-P group (P<0.001).

EMG of the anal sphincter revealed distinct neurogenic changes. The mean duration of motor unit potentials (MUPs) was significantly prolonged in the MSA-P group compared to PD (13.00 [11.90–13.90] ms vs. 10.90 [10.30–12.10] ms; P<0.001). Furthermore, the percentage of satellite potentials, indicative of reinnervation, was significantly higher in MSA-P patients [10.00 (5.00–15.00) %] compared to PD patients [4.85 (0.00–5.00) %; P = 0.001].

Notably, RBD was observed in a significantly larger proportion of the MSA-P cohort (78.38%) compared to the PD cohort (44.00%; P<0.001), highlighting the high frequency of RBD as a prodromal feature in α-synucleinopathies, particularly MSA.

### 3.2 Different distribution of α-Syn forms in erythrocytes and peripheral inflammatory signatures in the total cohort

**Figure 1:**
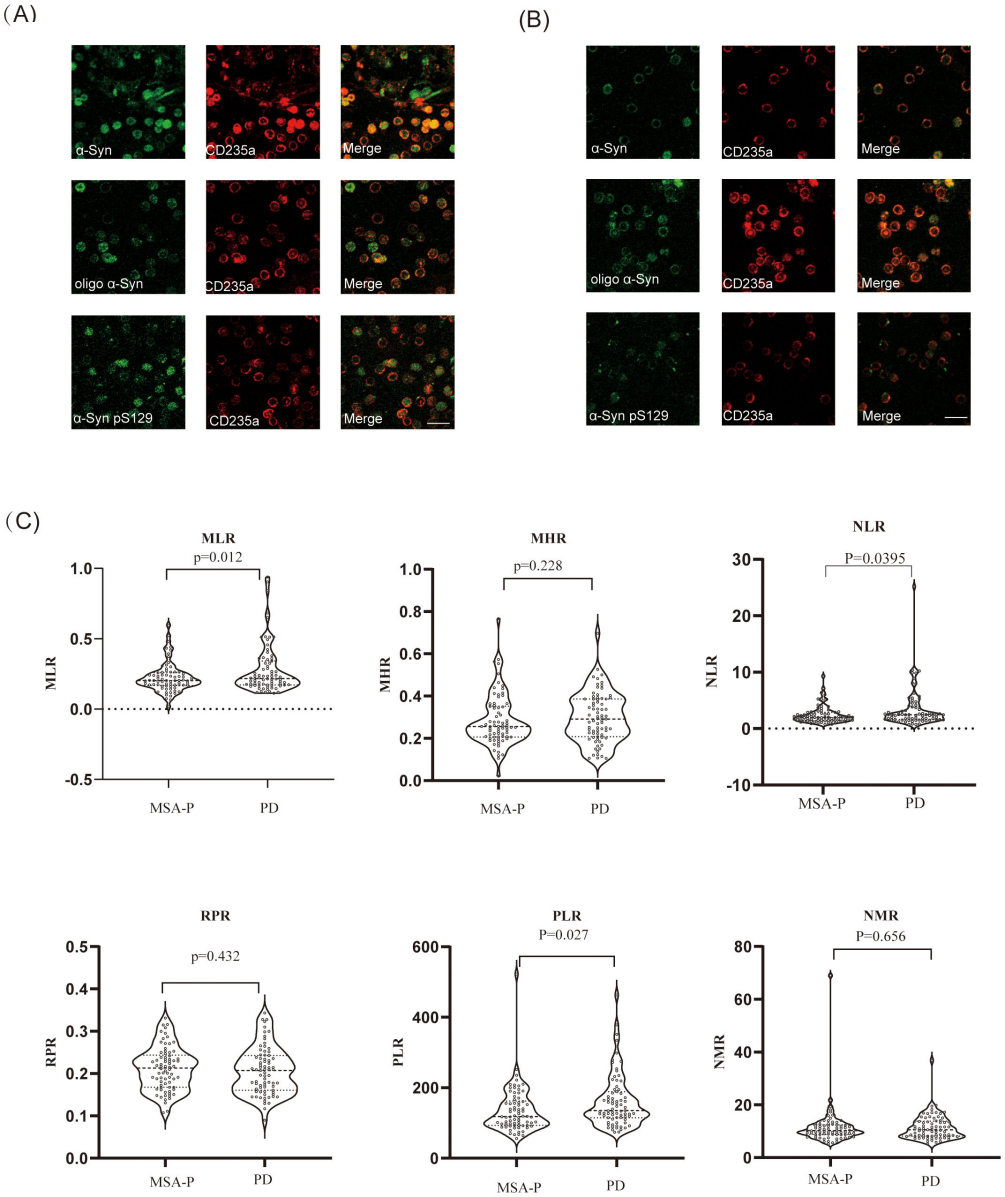
α-Synuclein distribution forms distribution in erythrocyte and peripheral inflammatory signatures in the total cohort of MSA-P and PD patients. Representative immunofluorescence images captured by a confocal microscope (40×). Distribution of α-Synuclein (α-Syn), oligomeric α-Synuclein, phosphorylated at Ser129 (pS129) α-Synuclein distribution pattern in erythrocytes of PD (n=10) **(A)** and MSA-P (n=10) **(B)** group. Green fluorescence indicates the specific alpha-Syn forms, while red fluorescence marks the erythrocyte membrane. Scale bars: 20 μm. **(C)** Box plots illustrating the comparison of derived peripheral blood inflammatory indices, including neutrophil-to-lymphocyte ratio (NLR), monocyte-to-lymphocyte ratio (MLR), and platelet-to-lymphocyte ratio (PLR). While markers like MHR and RPR showed no variation, PD patients (n=75) exhibited significantly elevated MLR (P = 0.012) and PLR (P = 0.027), whereas MSA-P patients (n=74) showed higher NLR (P = 0.0359), suggesting distinct inflammatory profiles in established disease stages. The independent t-test was applied for normally distributed continuous data, and the Mann–Whitney U test for non-normally distributed data when comparing two groups. A two-sided p-value < 0.05 was considered statistically significant. NLR, neutrophil-to-lymphocyte ratio; NMR, neutrophil-to-Monocyte Ratio; MLR, monocyte-to-lymphocyte ratio;PLR, Platelet-to-Lymphocyte Ratio; MHR, monocyte-to-HDL Ratio; RPR, red blood cell distribution width-to-platelet ratio.

We further characterized the distribution of three alpha-Syn forms (α-Syn, Oligo-α-Syn and α-Syn pS129) in erythrocytes via immunofluorescence. In PD patients, both total α-Syn and Oligo-α-Syn were detected in the cytoplasm and on the cell membrane (**Figure 1A**). In contrast, in MSA-P patients, these forms were predominantly distributed on the cell membrane fractions, with only minor presence in the cytoplasm (**Figure 1B**). While α-Syn pS129 was detected in both cellular compartments for both groups, it showed a preferential localization to the cytoplasm in PD patients. The demographic and hematological profiles of the patients are detailed in **Table 2**.

**Table 2:** Demographic and clinical characteristics of α-Syn forms in erythrocytes cohort (MSA-P vs. PD).

Characteristics	MSA-P(n=10)	PD(n=10)	P
Females/Males	6/4	5/5	0.739
Age, years	63.60 ± 3.03	65.00 ± 2.91	0.305
Duration, years	3.50 ± 1.65	5.40 ± 2.96	0.093
PVR urine volume, ml	102.30 ± 138.86	15.99 ± 28.58	**0.035**
HDL-C	1.35 ± 0.42	1.42 ± 0.25	0.648
Hcy	14.61 ± 6.34	24.59 ± 21.60	0.178
MMSE	27.00(26.00-29.00)	27 (25.50-29.25)	1.000
MoCA	21.50 ± 3.47	23.40 ± 4.03	0.274
1minΔSBP,mmHg	21.60 ± 13.53	3.30 ± 22.02	**0.038**
1minΔDBP,mmHg	10.00 ± 8.39	0.40 ± 13.58	0.073
3minΔSBP,mmHg	21.30 ± 13.61	2.40 ± 23.06	**0.039**
3minΔDBP,mmHg	8.90 ± 7.22	-1.80 ± 13.82	**0.042**
eGFR,ml/min	107.08 ± 7.06	106.18 ± 6.70	0.774
Constipation%	80.00	80	**0.003**
diabetes%	0.00	20.00	1.000
nephropathy%	0.00	0.00	1.000
hypertension%	40.00	10.00	0.280
RBD%	80.00	70.00	**0.739**
smoking%	30.00	30.00	1.000
Drinking%	20.00	30.00	1.000
Duration(ms)	13.41 ± 2.17	11.24 ± 1.25	**<0.001**
Polyphasity(%)	52.50(29.50-78.00)	40.00(30.00-52.50)	0.393
Percentage of satellite potentials(%)	12.50 ± 12.75	4.00 ± 3.16	0.056

PVR, post-void residual; MSA, Multiple system atrophy; PD, Parkinson’s Disease; HDL-C, high-density lipoprotein cholesterol; Hcy, homocysteine; ΔSBP, changes in systolic blood pressure during the supine-to-standing test. ΔDBP, changes in diastolic blood pressure during the supine-to-standing test; MMSE, Mini-Mental State Examination; MoCA, Montreal Cognitive Assessment.

Comparison of baseline demographic variables, clinical scales, and autonomic parameters between patients with Multiple System Atrophy-Parkinsonian variant (MSA-P, n=10) and Parkinson’s Disease (PD, n=10). Continuous variables were described as mean (SD) or median (IQR) according to data distribution, and categorical variables were reported as n (%). The t-test and the chi-square test were used to compare differences in clinical data between groups. The Mann–Whitney U test was used for non-normally distributed data between two groups. A two-sided p-value < 0.05 was considered statistically significant.

Bold values indicate statistically significant differences.

Given the emerging role of peripheral inflammation in neurodegenerative progression, we analyzed peripheral blood inflammatory indices derived from routine hematological parameters: NLR, LMR, MLR, MHR, RPR, and PLR. As illustrated in **Figure 1C**, while markers such as MHR (P = 0.228), RPR (P = 0.432), and NMR (P = 0.656) showed no significant variation between groups. The MLR, NLR and PLR were significantly elevated in the PD group compared to the MSA-P group (p<0.05). These findings suggest that PD patients may exhibit a peripheral pro-inflammatory state characterized by lymphocyte suppression or myeloid/platelet activation compared to MSA-P patients.

### 3.3 Absence of peripheral inflammatory changes in early-stage PD and possible MSA-P

To determine if the aforementioned inflammatory changes serve as early biomarkers, we performed a subgroup analysis on 36 early-stage PD and 38 possible MSA-P patients. Demographic and hematological characteristics are detailed in **Table 3**.

**Table 3:** Demographic and hematological characteristics of the early-stage groups.

Characteristics	Possible MSA-P (n=38)	Early-stage PD (n=36)	P
Leukocyte count(x 10^9^/L)	5.77(4.58-6.40)	5.24(4.41-6.09)	0.249
Neutrophils(x10^9^/L)	3.46(2.59-3.96)	2.98 (2.54-3.92)	0.378
Lymphocytes(x10^9^/L)	1.63(1.26-2.07)	1.65(1.42-1.87)	0.812
Eosinophils(x10^9^/L)	0.17 ± 0.14	0.15 ± 0.14	0.527
Basophils (x 10^9^/L)	0.03 ± 0.02	0.03 ± 0.02	0.407
Monocytes (x 10^9^/L)	0.33(0.27-0.42)	0.31(0.25-0.39)	0.156
PLT (x 10^9^/L)	198.00(167.25-249.00)	204.00(171.25-268.00)	0.499
MPV (fl)	9.48 ± 1.04	9.61 ± 1.16	0.607
RDW	41.80(40.58-43.65)	40.95(40.50-43.60)	0.496
1minΔSBP,mmHg	15.50(4.00-22.75)	-1.00(-8.00-12.00)	**0.003**
1minΔDBP,mmHg	3.00(-1.00-8.00)	-0.50(-10.00-3.75)	**0.004**
3minΔSBP,mmHg	11.00(2.00-18.50)	2.50(-4.75-10.00)	**0.008**
3minΔDBP,mmHg	2.50(-2.00-8.25)	-1.50(-7.00-3.75)	**0.008**
HDL-C	1.28(1.15-1.49)	1.24(1.05-1.65)	0.677
Hcy	14.78 ± 7.30	15.65 ± 8.54	0.633
NLR	2.42 ± 1.57	2.05 ± 0.91	0.226
NMR	11.81 ± 9.97	10.40 ± 3.37	0.422
MLR	0.23 ± 0.12	0.20 ± 0.07	0.146
PLR	137.30 ± 48.17	137.21 ± 48.53	0.994
MHR	0.25(0.21-0.35)	0.24(0.20-0.31)	0.449
RPR	118.05(98.64-177.78)	0.20(0.16-0.25)	0.516

HDL-C, high-density lipoprotein cholesterol; Hcy, homocysteine; NLR, neutrophil-to-lymphocyte ratio; NMR, neutrophil-to-Monocyte Ratio; MLR, monocyte-to-lymphocyte ratio; PLR, Platelet-to-Lymphocyte Ratio; MHR, monocyte-to-HDL Ratio; RPR, red blood cell distribution width-to-platelet ratio.

Comparison of routine blood indices and derived inflammatory ratios between patients with Possible MSA-P (n=38) and Early-stage PD (n=36). Unlike the total cohort, no statistically significant differences were found in leukocyte counts, neutrophil-to-lymphocyte ratio (NLR), or other inflammatory indices (MLR, PLR) at this early clinical stage (P > 0.05 for all), highlighting the need for higher-resolution analysis. Data are shown as the mean (SD) for normally distributed continuous variables, median (IQR) for nonnormally distributed continuous variables. The t-test and the chi-square test were used to compare differences in clinical data between groups. The Mann–Whitney U test was used for non-normally distributed data between two groups. A two-sided p-value < 0.05 was considered statistically significant.

Bold values indicate statistically significant differences.

In contrast to the total cohort, the early-stage comparison revealed no statistically significant differences across routine hematological counts or derived inflammatory indices. Leukocyte, neutrophil, lymphocyte, and monocyte counts were comparable between groups (P>0.05). Crucially, the indicators that distinguished the groups in the full cohort lost their discriminative power in the early stages: NLR (P = 0.226), MLR (P = 0.146), and PLR (P = 0.994) showed no significant variance. This indicates that standard systemic inflammatory indices lack the sensitivity to distinguish between PD and MSA-P during early clinical phases.

### 3.4 Single-cell sequencing of monocyte alterations

The absence of gross hematological differences prompted us to employ high-resolution scRNA-seq to investigate subtle transcriptomic alterations masked in bulk analyses. We analyzed PBMCs from 3 healthy controls (HC), 3 early-stage PD patients, and 3 possible MSA-P patients. Baseline demographic and clinical characteristics are summarized in **Table 4**.

**Table 4:** Demographic and clinical characteristics of single-cell sequencing study cohort.

Characteristics	HC group (n=3)	Possible MSA-P(n=3)	Early-stage PD (n=3)	P
Females/Males	3/0	2/1	1/2	
Age, years	60 [min 58, max 62]	60.33 [min 58, max 60]	62.67 [min 59, max 68]	0.729
Duration, years	/	2.00 [min 1.00, max 3.00]	3.00 [min 1.00, max 3.00]	0.70
Leukocyte count(x 10^9^/L)	/	1.57 [min 0.42, max 3.20]	0.55 [min 0.00, max 1.44]	0.40
Neutrophils(x10^9^/L)	/	5.31 [min 4.44, max 6.50]	6.61 [min 4.32, max 9.06]	0.10
Lymphocytes(x10^9^/L)	/	1.87 [min 1.35, max 2.16]	2.11 [min 0.80, max 3.58]	0.10
UA	/	241.70 [min 186.80, max 269.80]	279.1 [min 264.30, max 301.60]	0.40
PVR urine volume, ml	/	170.33 [min 127, max 234]	29.67 [min 18.00, max 46.00]	0.10
HDL-C	/	1.39 [min 1.26, max 1.60]	1.49 [min 1.32, max 1.73]	0.40
Hcy	/	11.29 [min 9.82, max 12.81]	14.37 [min 11.46, max 15.90]	0.20
MMSE	/	27.00 [min 25.00, max 29.00]	28.00 [min 26.00, max 30.00]	0.70
MoCA	/	22.00 [min 19.00, max 24.00]	26.33 [min 25.00, max 28.00]	0.10
1minΔSBP, mmHg	/	34.33 [min 10.00, max 62.00]	4.33 [min 1.00, max 8.00]	0.10
3minΔSBP, mmHg	/	39.00 [min 9.00, max 24.00]	-1.00 [min -19.00, max 8.00]	0.10

PVR, post-void residual; MSA, Multiple system atrophy; PD, Parkinson’s Disease; HDL-C, high-density lipoprotein cholesterol; Hcy, homocysteine; ΔSBP, changes in systolic blood pressure during the supine-to-standing test. ΔDBP, changes in diastolic blood pressure during the supine-to-standing test; MMSE, Mini-Mental State Examination; MoCA, Montreal Cognitive Assessment.

Comparison of baseline demographic variables, clinical scales among patients with Healthy control (n=3), Multiple System Atrophy-Parkinsonian variant (possible MSA-P, n=3) and early-stage Parkinson’s Disease (PD, n=3). Continuous variables are presented as mean [minimum, maximum], and categorical variables are shown as counts. The Mann–Whitney U test was used for pairwise comparisons between the possible MSA-P and early-stage PD groups. The Kruskal–Wallis H test was applied to compare continuous variables among the three groups. Due to the small sample size, gender distribution was only described qualitatively. Statistical significance was defined as a two-tailed p < 0.05.

**Figure 2:**
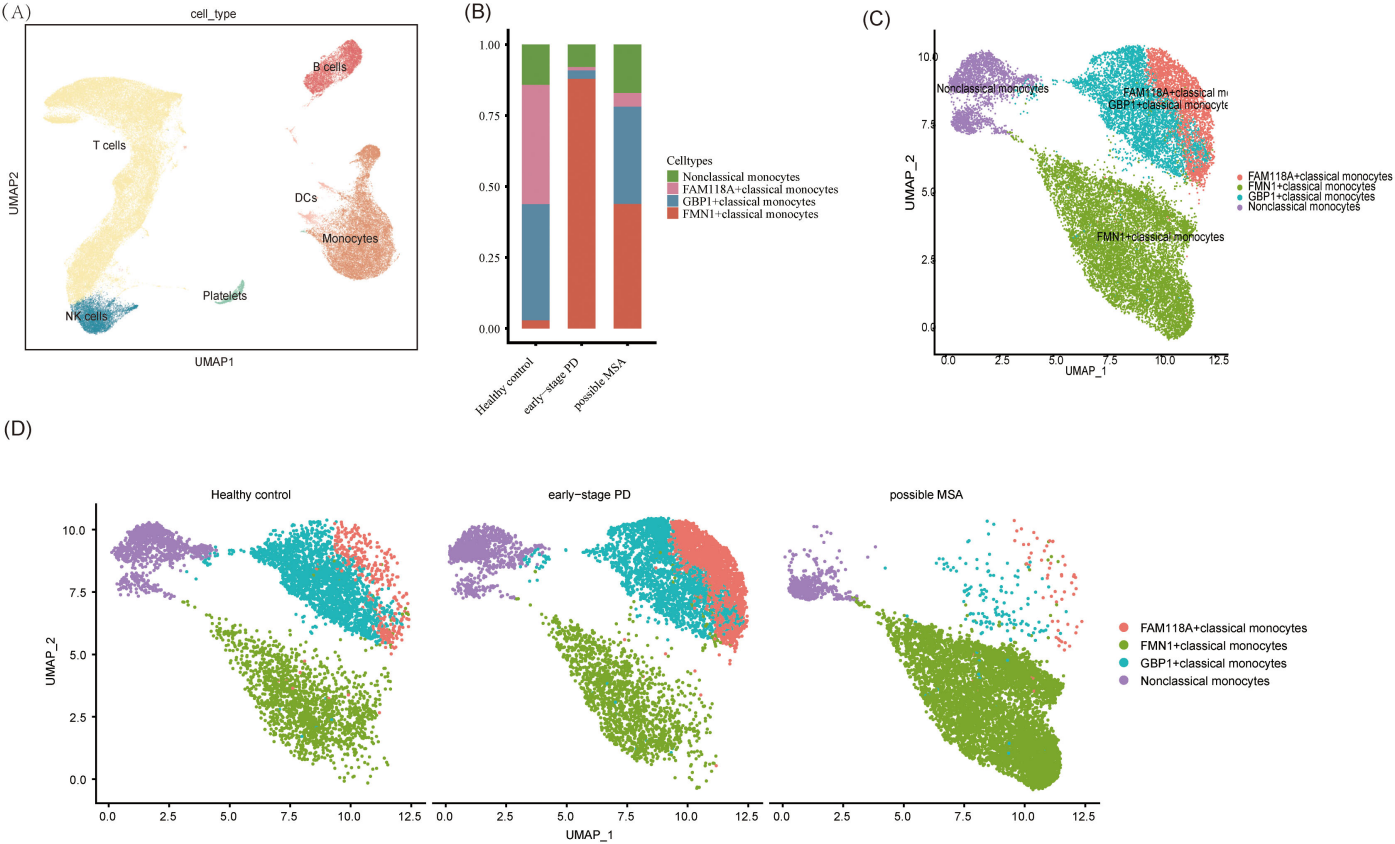
Single-cell resolution of peripheral immune landscape and monocyte alterations in early-stage neurodegeneration. **(A, B)** Cellular composition analysis showing the distribution and proportion of immune cell subsets (T cells, B cells, NK cells, DCs, Monocytes) across Healthy Controls (HC) (n=3), Early-stage PD (n=3), and Possible MSA-P(n=3). Note the expansion of Classical Monocytes in disease groups. **(C)** UMAP visualization identifying major monocyte subtypes: FAM118A+ classical, N1+ classical, GBP1+ classical, and non-classical monocytes. **(D)** Comparative distribution analysis of these four monocyte subtypes across the three study groups.

We first examined cellular composition (**Figures 2A, B**). The UMAP and proportion of various immune cell subsets among the three groups—including T cells (CD4+, CD8+), B cells, NK cells, and DCs—was quantified. Notably, we observed a distinct expansion in the proportion of Classical Monocytes in both early-stage PD and possible MSA-P groups compared to healthy controls. This suggests that classical monocyte expansion is a shared systemic response to incipient neurodegeneration. Sub-clustering identified specific monocyte populations, including FAM118A+, FMN1+, and GBP1+ classical monocytes, alongside non-classical monocytes (**Figure 2C**). The distribution of these four cell types was further compared across the three groups (**Figure 2D**).

### 3.5 Differential gene expression and enrichment analysis

We next sought to identify specific gene expression changes distinguishing early-stage PD from possible MSA-P via DGE analysis. The resulting volcano plots highlight significantly upregulated and downregulated genes.

**Figure 3:**
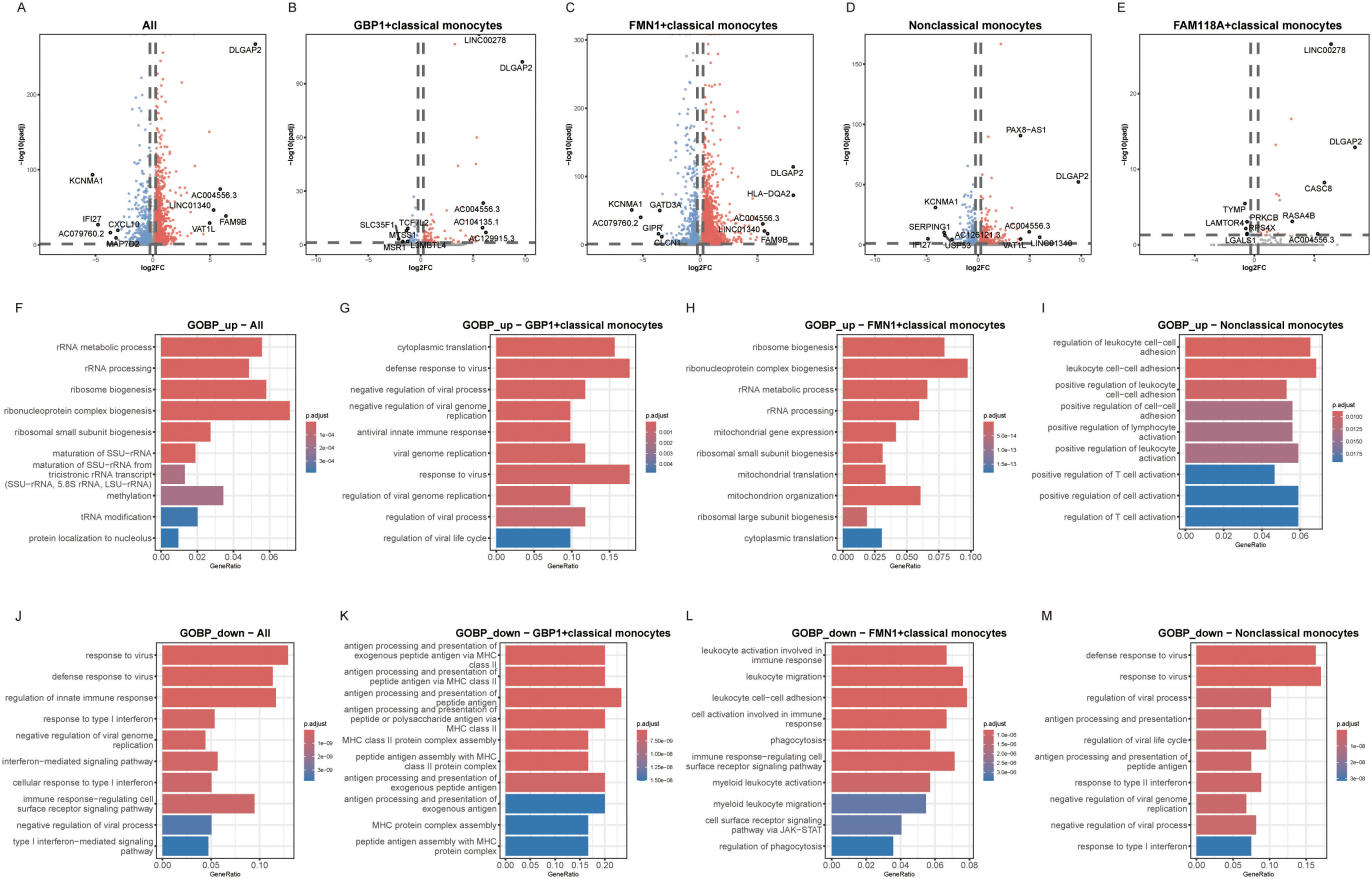
Differential gene expression (DGE) analysis of monocyte distinguishing early-stage PD from possible MSA-P. Volcano plots illustrating significantly upregulated and downregulated genes in specific monocyte subpopulations. **(A)** total monocytes; **(B)** GBP1+ classical monocyte subpopulation; **(C)** FMN1 subpopulation; **(D)** FAM118A+ classical monocyte subpopulation; and **(E)** non-classical monocyte subpopulation. GO Enrichment Analyses of Differentially Expressed Genes (DEGs) in Monocytes from Early-Stage PD (n=3) and Possible MSA-P Group (n=3). **(F, J)** the upregulated and down regulated DEGs in monocytes; **(G, K)** the upregulated and downregulated DEGs in GBP1+ subpopulation; the upregulated and downregulated DEGs in FMN1 classical monocyte subpopulation **(H, L)** and non-classical monocyte subpopulation **(I, M)**.

After excluding sex-chromosome-related genes (e. g., RPS4Y1, XIST), distinct signatures emerged. In possible MSA-P compared with early-stage PD (**Figure 3A**), the upregulation genes were chemokines CXCL10, CXCL5, GATD3A,MAP7D2 and IFI27. Conversely, downregulated genes included DLGAP2 and PAX8-AS1.In the GBP1 subpopulation, non-coding region transcripts AC004556.3 and AC104135.1 were upregulated in early-stage PD (**Figure 3B**). In the FMN1 subpopulation (**Figure 3C**), HLA-DQA2 and LINC01340 were upregulated in early-stage PD group compared to possible MSA-P group. In the FAM118A subpopulation, LINCOO278 and DLGAP2 were upregulated in early-stage PD group (**Figure 3E**). The PAX8-AS1 and non-coding region transcripts AC004556.3 were upregulated in the nonclassical monocytes subpopulation compared with early-stage PD to possible MSA-P patients (**Figure 3D**).

GO analysis of upregulated DEGs in early-stage PD were associated with “RNA metabolism process”, “ribosome biogenesis” and “rRNA process” pathways (**Figure 3F**). Conversely, genes upregulated in MSA-P (downregulated in PD) focused on “response to virus”, “regulation of innate immune response” and “response to interferon-mediated signaling”(**Figure 3J**). In addition, the upregulated DEGs in GBP1 subpopulation revealed associations with “cytoplasmic translation” (**Figure 3G**) and the downregulated DEGs were mainly associated with “antigen processing and presentation of peptide antigen via MHC class II” (**Figure 3K**). The upregulated DEGs in FMN1 subpopulation were enriched for “ribosome biogenesis” (**Figure 3H**), whereas the corresponding downregulated DEGs were associated with “leukocyte activation involved in immune response, migration and cell-cell adhesion.” (**Figure 3L**). The upregulated DEGs in the non-classical monocyte subpopulation were associated with the “regulation of leukocyte cell-cell adhesion” (**Figure 3I**), while the downregulated DEGs were enriched in “defense response to virus” and “antigen processing and presentation” (**Figure 3M**).

### 3.6 KEGG enrichment analyses of DEGs in monocytes from early-stage PD and possible MSA-P group

**Figure 4:**
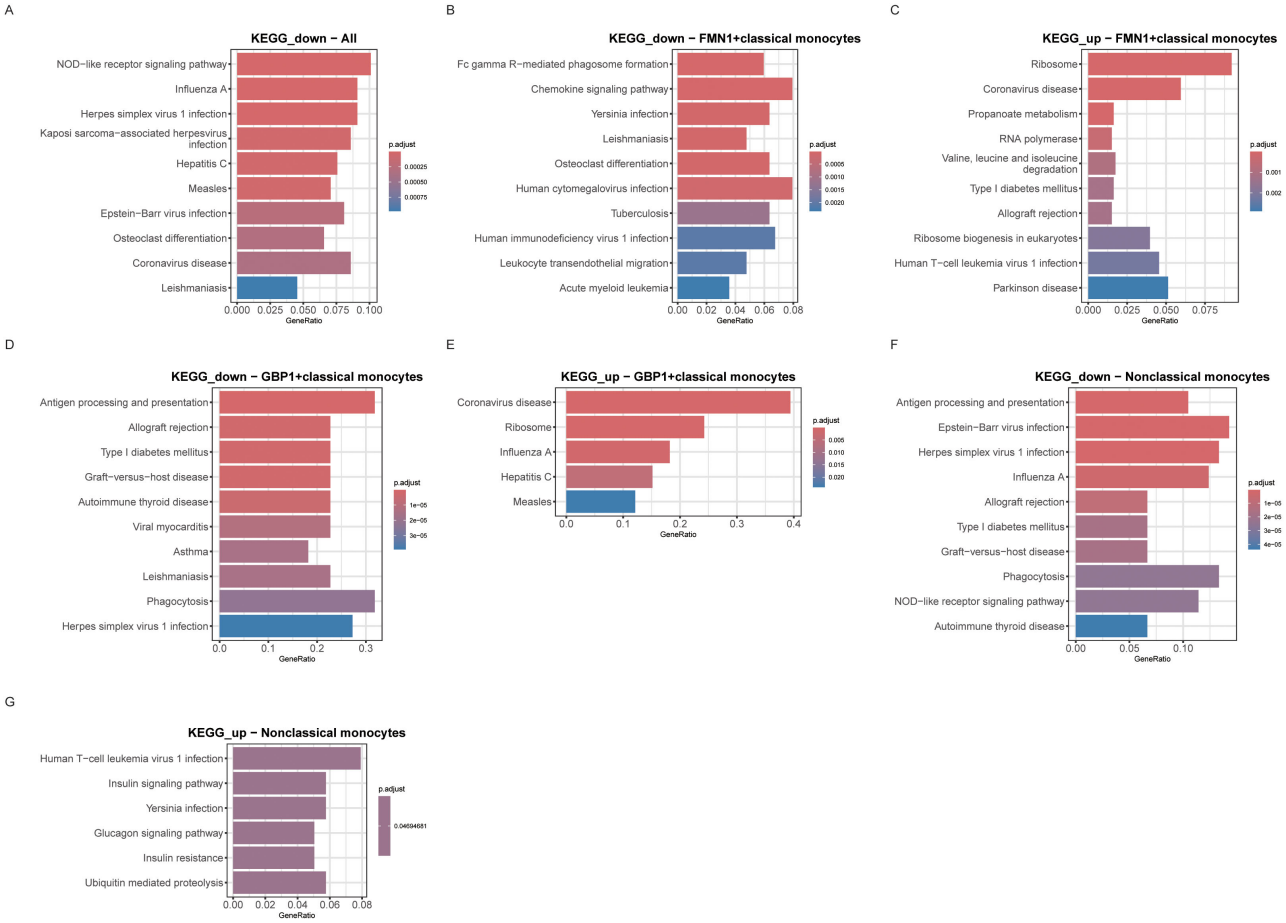
KEGG enrichment analyses of DEGs in monocytes from early-stage PD and possible MSA-P group. **(A)** total monocytes (downregulated); **(B, C)** FMN1 subpopulation (downregulated and upregulated); **(D, E)** GBP1+ classical monocyte subpopulation (downregulated and upregulated); and **(F, G)** non-classical monocyte subpopulation (downregulated and upregulated). Early-stage PD (n=3) and possible MSA-P group (n=3).

KEGG analysis identified that downregulated DEGs were enriched in “NOD-like receptor signaling pathway”, “Coronavirus disease” and “antigen processing and presentation” in early-stage PD (**Figure 4A**). Additionally, the downregulated DEGs in FMN1 subpopulation were also associated with “Fc gamma R-mediated phagosome formation” (**Figure 4B**). Downregulated DEGs were primarily mapped to “antigen processing and presentation” in in GBP1 subpopulation (**Figure 4D**) and nonclassical monocytes(**Figure 4F**). While the upregulated DEGs were involved in “Coronavirus disease - COVID-19” and “Human T-cell leukemia virus 1 infection” (**Figures 4C, E, G**).

### 3.7 GSVA score and cell-cell communication networks

To link transcriptomic changes to broader pathophysiological mechanisms, we performed Gene Set Variation Analysis (GSVA).

**Figure 5:**
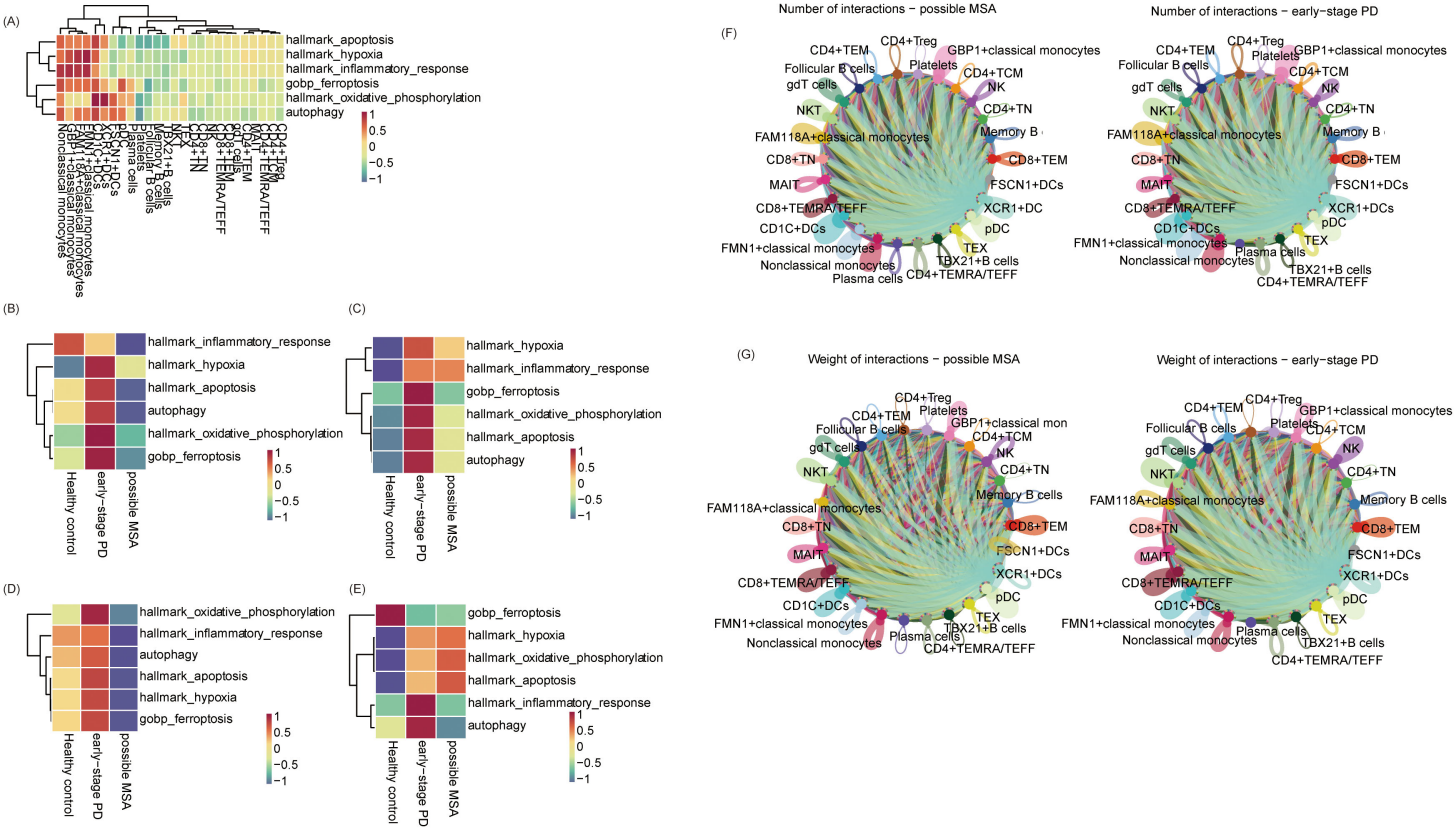
GSVA score and alterations in cell-cell communication networks. **(A)** Heatmap exhibiting enrichment scores for key pathways (Ferroptosis, Oxidative Phosphorylation, Autophagy, Apoptosis, Hypoxia, Inflammatory Response) across different cell types. **(B-E)** Enrichment scores comparing Early-stage PD (n=3) and Possible MSA-P (n=3) across subtypes: **(B)** GBP1+ classical monocytes; **(C)** FMN1 subpopulation; **(D)** FAM118A+ classical monocytes; and **(E)** non-classical monocytes. **(F, G)** Netplots visualizing the number and strength of intercellular interactions in the early-stage PD group (n=3) and the possible MSA-P group (n=3).

As shown in the heatmap, the upregulated signaling pathway such as “Hypoxia” and “Inflammatory Response” suggest a compromised capacity of peripheral monocytes response, indicating that monocytes are closely involved in the pathomechanism of Parkinsonism (**Figure 5A**).

Comparing early-stage PD group with possible MSA-P group, the GSVA score revealed that the autophagy and apoptosis pathways were highlighted in GBP1 subpopulation (**Figure 5B**), and oxidative phosphorylation, apoptosis, hypoxia and ferroptosis pathway were upregulated in the FMN1 and FAM118A subpopulation (**Figures 5C, D**). The Oxidative phosphorylation, hypoxia and ferroptosis pathway were upregulated in nonclassical monocytes subpopulation (**Figure 5E**).

Finally, CellChat analysis was employed to infer intercellular communication. In the early-stage PD group, the number and weight of intercellular interactions were higher than in the MSA-P group, suggesting a more active communication network (**Figures 5F, G**). The number and strength of interactions between monocyte subsets and other cell types also exhibited consistent alterations in the two groups.

### 3.8 Validation of gene expression via qRT-PCR

**Figure 6:**
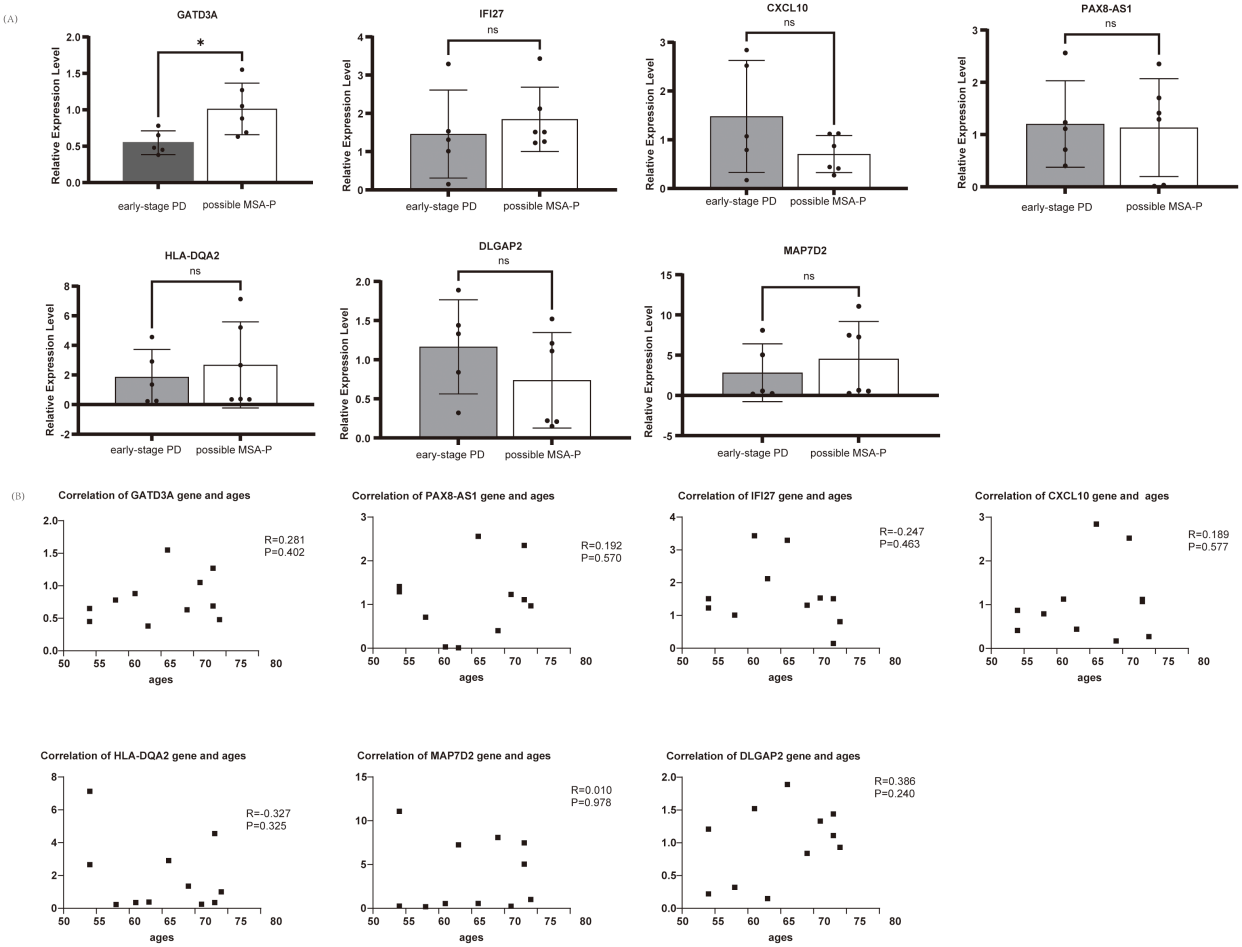
Validation of screened genes expression monocytes between early-stage PD and possible MSA-P patients by qRT-PCR, along with analysis of the correlation between these genes and ages. GAPDH was used as the internal reference gene. Each dot represents an individual biological replicate. **(A)** Differential analysis of screened genes between early-Stage PD (n=5) and possible MSA-P group(n=6); The t-test was used to compare differences in clinical data between groups, *P < 0.05. **(B)** Correlation analysis of screened differentially expressed genes with ages. Comparison of relative gene expression levels in monocytes between the two groups. Pearson correlation analysis was performed to evaluate the correlation between gene expression levels and patient age.

We utilized qRT-PCR to validate the expression of screened genes in peripheral blood monocytes. Baseline demographic and clinical characteristics are summarized in **Table 5**. Consistent with the scRNA-seq results, GATD3A expression was significantly upregulated in possible MSA-P compared to early-stage PD. Another gene, IFI27, showed an increasing trend in possible MSA-P, though the difference did not reach statistical significance in this validation cohort. Other candidate genes showed no statistically significant differences (**Figure 6A**). In addition, we also performed correlation analyses between the expression levels of GATD3A (and other validated genes) and patient age (**Figure 6B**). The results showed no significant correlation between gene expression levels and age, demonstrating that the observed differences between early- stage PD and possible MSA-P are independent of age.

**Table 5:** Demographic and clinical characteristics of validation cohort.

Characteristics	Possible MSA-P (n=6)	Early-stage PD (n=5)	P
Females/Males	3/3	2/3	0.740
Age, years	62(54.00-69.50)	69.00(62.00-72.00)	0.329
Duration, years	2.00(2.00-3.25)	5.00(4.00-7.00)	**0.009**
PVR urine volume, ml	78.30(0.00-235.00)	12.00(0.00-22.50)	0.247
MMSE	27.00(21.50-29.25)	23.00(22.00-27.50)	0.662
MoCA	25.50(11.25-26.50)	22.00(16.00-25.50)	0.662
1minΔSBP,mmHg	5.00(0.00-34.50)	0.00(-5.00-15.00)	0.177
3minΔSBP,mmHg	7.50(-2.75-49.00)	-4.00(-11.50-17.00)	0.247

PVR, post-void residual; MSA, Multiple system atrophy; PD, Parkinson’s Disease; ΔSBP, changes in systolic blood pressure during the supine-to-standing test; MMSE, Mini-Mental State Examination; MoCA, Montreal Cognitive Assessment.

Comparison of baseline demographic variables, clinical scales between patients with possible Multiple System Atrophy-Parkinsonian variant (possible MSA-P, n=6) and early-stage Parkinson’s Disease (early-stage PD, n=5). Data are shown as median (IQR) for nonnormally distributed continuous variables, and categorical gender data were presented as frequencies. The Mann–Whitney U test or chi-square test was used between two groups. A two-sided p-value < 0.05 was considered statistically significant.

Bold values indicate statistically significant differences.

## 4 Discussion

Our clinical cohort analysis corroborates previous findings (28, 29), confirming that MSA-P is associated with a markedly shorter disease duration, a more rapid trajectory of disability accumulation, and a distinct profile of autonomic dysfunction compared to PD (30, 31). Approximately 60% of MSA patients experience urinary system disorders prior to or concurrently with motor dysfunction, with 18.2% presenting these as the initial symptom (32). Consistent with MDS criteria identifying PVR ≥100 mL as a critical marker, our data reveal significantly higher PVR in MSA-P compared to PD (p < 0.001). Furthermore, electromyography (EMG) of the external anal sphincter in MSA-P patients showed significantly increased duration, phases, polyphasicity, and satellite potential prevalence, indicative of severe neurogenic damage. Cardiovascular autonomic failure was also more pronounced; while orthostatic hypotension (OH) typically manifests in advanced PD (20%–60%), it occurs in over 60% of MSA patients upon active standing (33, 34). Our study indicates that ΔSBP (at 1 and 3 minutes) are significantly elevated in MSA-P compared to PD. Notably, while rapid eye movement sleep behavior disorder (RBD) is a hallmark of α-synucleinopathies (35), its prevalence was significantly higher in our MSA-P cohort (78.38%) than in the PD cohort (44.00%), highlighting its utility as a diagnostic “red flag” (36).

Beyond clinical phenotypes, our investigation highlights divergent pathological protein distributions. Building on our prior work correlating erythrocytic alpha-Syn levels with PD progression, we identified distinct compartmentalization patterns (8). In MSA-P, alpha-Syn and its oligomers are predominantly sequestered on the erythrocyte membrane with minimal cytoplasmic presence, whereas in PD, they are distributed across both compartments. Moreover, α-Syn pS129 preferentially localizes to the cytoplasm in PD but is ubiquitous in MSA-P. We postulate that toxic α-Syn species on the erythrocyte membrane in MSA may compromise membrane integrity and cellular function, thereby facilitating the intercellular transmission and propagation of α-Syn—a potential driver of the rapid disease progression observed in MSA. Thus, the differential subcellular distribution of erythrocytic alpha-Syn serves as a promising discriminatory indicator.

A central aim was to assess peripheral inflammatory markers. While literature suggests elevated intermediate monocytes and activation markers correlate with PD severity, and higher MHR levels indicate severe inflammation in MSA, our findings paint a nuanced picture (15, 37, 38). In our total cohort, MLR, NLR, and PLR were significantly higher in PD patients than MSA-P group. We speculate that in MSA-P, peripheral immune cells may infiltrate the CNS to a greater extent, leaving fewer circulating cells and driving a more intense central neuro-inflammatory responses (39). However, we noted a discrepancy in the second cohort: patients with early-stage PD and possible MSA-P exhibited significant differences in orthostatic SBP and DBP fluctuations, yet no significant intergroup differences were found in peripheral inflammatory ratios (MLR, NLR, and PLR). Based on the literature, several confounding factors may explain this lack of correlation. First, as reported by Li et al. (40), inflammation mediated by phosphorylated α-synuclein/TLR2 signaling at this stage may be localized to vagal Schwann cells rather than triggering widespread systemic immune activation. Furthermore, systemic inflammatory responses typically become pronounced only during advanced stages of the disease. Second, concurrent medications represent a major confounding factor; both dopaminergic therapies and common antihypertensive drugs have documented anti-inflammatory properties that can modulate circulating inflammatory cell profiles (41). Variations in drug classes and dosages between the two cohorts may therefore mask subtle inflammatory differences associated with orthostatic blood pressure fluctuations. Third, individual variations in residual vagal cardiac modulation—a key component of the cholinergic anti-inflammatory pathway—could counteract peripheral inflammation to varying degrees. This compensatory buffering mechanism may homogenize peripheral inflammatory levels across patient subgroups, despite their divergent blood pressure dysregulation (42). Finally, baseline heterogeneity, including age distribution and chronic comorbidities, can also affect the stability of circulating inflammatory markers. Collectively, these factors may account for the inconsistent findings in our cohort: while composite inflammatory markers correlate with disease severity (43), they lack the sensitivity required for early differential diagnosis.

The limitations of bulk markers prompted us to employ scRNA-seq and qRT-PCR to dissect subtle early-stage immune alterations. scRNA-seq unveiled a shared expansion of classical monocytes in both early PD and MSA-P, indicating a common innate immune response to incipient neurodegeneration. However, the transcriptomic landscapes diverged significantly. Early MSA-P monocytes exhibited an “aggressive recruitment” phenotype, evidenced by the upregulation of chemokines CXCL10 and CXCL5. CXCL10 facilitates trans-endothelial migration, aligning with neuropathological reports of extensive lymphocyte infiltration in MSA brains (44). Furthermore, upregulation of IFI27 points to interferon pathway activation in MSA-P, which can exert neurotoxic effects when chronically activated (45). GATD3A is an essential mitochondrial deglycase that limits the accumulation of advanced glycation end products and preserves mitochondrial homeostasis. The elevation of GATD3A in MSA-P likely reflects unremitting mitochondrial damage and compensatory stress responses triggered by intense oxidative and glycative stress. In contrast, early-stage PD monocytes displayed enhanced transcriptional regulation and neuronal support (e.g., *DLGAP2*, HLA-DQA2). Validated by qRT-PCR, GATD3A expression was significantly upregulated in possible MSA-P compared to early-stage PD. Although IFI27 showed an upward trend in possible MSA-P and DLGAP2 in PD, these did not reach statistical significance in the validation cohort, warranting larger studies. The selective upregulation of GATD3A in possible MSA-P suggests a heightened demand for maintaining mitochondrial integrity. Integrating the expression pattern of GATD3A with GO enrichment results indicates that possible MSA-P is characterized by robust activation of innate immunity and interferon-mediated signaling pathways. The compensatory upregulation of GATD3A points to severe mitochondrial stress, while persistent immune activation likely exacerbates pathological damage, contributing to the more aggressive clinical progression of MSA-P. Conversely, the activation of pathways related to RNA metabolism, ribosome biogenesis, and cytoplasmic translation in early-stage PD, alongside the downregulation of GATD3A, reflects a state of metabolic reprogramming aimed at maintaining mitochondrial homeostasis while limiting excessive immune activity.

GSVA analysis further characterized these populations as being enriched in “hypoxia”, “ferroptosis”, and “inflammatory response” pathways. This implies that peripheral monocytes in synucleinopathies are not quiescent but exist in a state of metabolic stress, consistent with BBB disruption allowing peripheral sensing of central pathology. Early PD monocytes exhibited enhanced oxidative phosphorylation and apoptosis signaling, consistent with GO results showing that epigenetic methylation and mitochondrial translation support hypoxia resistance and suppress excessive inflammation.

CellChat analysis linked these functional differences to immune regulation. Early PD had far more abundant ligand-receptor crosstalk than possible MSA-P (8,587 vs. 7,312 interactions; total weight: 229.237 vs. 163.262). Robust intercellular signaling in early-stage PD coordinates immune homeostasis, relieves metabolic stress, and restrains hyperactive innate/interferon responses-matching its lower GATD3A levels. By contrast, possible MSA-P monocytes displayed limited cell communication alongside severe mitochondrial oxidative and glycative damage, marked by compensatory GATD3A elevation. Deficient ligand-receptor feedback fails to curb persistent chemokine and interferon signaling, amplifying neuroinjury and accelerating MSA-P progression.

### 4.1 Limitations

Certain limitations warrant consideration. First, the sample size of our scRNA-seq cohort was relatively small; while sufficient to identify significant transcriptomic differences, validation in larger, multi-center cohorts is essential. Second, data were collected from different patient cohorts, which limited combined multivariate analysis. Third, this study focused on peripheral blood; correlating these findings with cerebrospinal fluid or post-mortem brain tissue would reinforce the link between peripheral immunity and central neuroinflammation. Future research should also map the longitudinal evolution of these immune features to determine their stability over the disease course.

## 5 Conclusion

In summary, while standard peripheral inflammatory markers fail to differentiate these synucleinopathies in their early stages, distinct transcriptomic alterations in monocytes are already detectable. In possible MSA-P, monocytes exhibit severe mitochondrial stress (marked by compensatory GATD3A upregulation) coupled with an aggressive, chemokine-driven interferon response, aligning with the rapid clinical progression. Conversely, early-stage PD monocytes display a profile geared toward homeostatic preservation and regulated immune activity. These divergent signatures, combined with the distinct subcellular distribution of erythrocytic alpha-Syn, provide new insights into the pathophysiological differences between these disorders and offer a framework for the development of early diagnostic biomarkers.

## Data Availability

The datasets presented in this study can be found in online repositories. The names of the repository/repositories and accession number(s) can be found in the article/**Supplementary Material**.

## Supplementary material

Supplementary Table 1 DNA sequences of the primers used in this study. The primers of seven screened genes were listed in the table.
