# Correction: GATD3A-mediated monocyte homeostasis and compartmentalized erythrocyte alpha-synuclein discriminate early Parkinson’s disease from MSA-P

**DOI:** 10.3389/fimmu.2026.1934828

**Published:** 2026-07-20

**Authors:** Ying Jiang, Chengcheng Xu, Jianing Jin, Xin Liu, Huihui Cai, Wanyu Zhang, Xiaoqing Zheng, Jiayi Wu, Zhan Wang, Yingshan Piao, Tao Feng

**Affiliations:** 1 Center for Movement Disorders, Department of Neurology, Beijing Tiantan Hospital, Capital Medical University, Beijing, China; 2 China National Clinical Research Center for Neurological Diseases, Beijing, China; 3 Department of Child and Adolescent Mental & Behavioral Health, Mental Health Center, Fifth People’s Hospital, ZiBo, Shandong, China; 4 Department of Neurology, Affiliated Hospital of Qingdao University, Qingdao, China; 5 Nursing Department of Beijing Tiantan Hospital, Capital Medical University, Beijing, China; 6 Capital Medical University, Beijing, China

**Keywords:** classical monocytes, multiple system atrophy, Parkinson’s disease, peripheral immune cells, single-cell RNA sequencing

Affiliation “China National Clinical Research Center for Neurological Diseases, Beijing, China” was erroneously given as “Department of Child and Adolescent Mental & Behavioral Health, China National Clinical Research Center for Neurological Diseases, Beijing, China”.

Affiliation “Department of Child and Adolescent Mental & Behavioral Health, Mental Health Center, Fifth People’s Hospital, ZiBo, Shandong, China” was erroneously given as “Mental Health Center, Fifth People’s Hospital, ZiBo, Shandong, China”.

