# Supplementary material for: GATD3A-mediated monocyte homeostasis and compartmentalized erythrocyte alpha-synuclein discriminate early Parkinson’s disease from MSA-P
Source: Front Immunol. 2026 Jul 9;17:1818028. doi: 10.3389/fimmu.2026.1818028 (PMC13431448; doi:10.3389/fimmu.2026.1818028)
Supplement: Supplementary Table 1 — DNA sequences of the primers used in this study. The primers of seven screened genes were listed in the table. [file Table1.docx]

**Supplementary Table 1 DNA sequences of the primers used in this study**

| **Name** | **DNA sequences of primers** |
| --- | --- |
| DLGAP2 | Forward: CTGCTCCTCCATCGGGATTC  Reverse: TCATCTTCCACTTGCACCCC |
| HLA-DQA2 | Forward: TCCAGTAACACAGGAGCAACC  Reverse: AAGGGCAGAGGGTTCGTTG |
| PAX8-AS1 | Forward: CCATGCCTCCCTCTCCTACT  Reverse: TGCCTTCGTGCTGACATTCT |
| CXCL10 | Forward: AGCAGAGGAACCTCCAGTCT  Reverse: TGCAGGTACAGCGTACAGTT |
| MAP7D2 | Forward: GACCTCTCAGCCCAACTACC  Reverse: CTCCAGGATCTGTTGCTCCC |
| IFI27 | Forward: CACATCCAAGCTTAAGACGGT  Reverse: ATTCCGTGGCATTCCAGAGTC |
| GATD3A | Forward: TCTGCATTCACGTCCCTGTC  Reverse: CGTAGACTCCGCATCCAGAC |
| GAPDH | Forward:ACAACTTTGGTATCGTGGAAGG  Reverse: GCCATCACGCCACAGTTTC |
